# Dextran as internal calibrant for *N*-glycan analysis by liquid chromatography coupled to ion mobility-mass spectrometry

**DOI:** 10.1007/s00216-022-04133-0

**Published:** 2022-05-26

**Authors:** Christian Manz, Michael Götze, Clemens Frank, Andreas Zappe, Kevin Pagel

**Affiliations:** 1 Department of Molecular Physics, Fritz Haber Institute of the Max Planck Society, Faradayweg 4-6, 14195 Berlin, Germany; 2 Department of Chemistry and Biochemistry, Freie Universität Berlin, Altensteinstr. 23A, 14195 Berlin, Germany; 3 Present Address: Analytical Chemistry, CMC, Silence Therapeutics GmbH, Robert-Rössle-Str. 10, 13125 Berlin, Germany

**Keywords:** N-Glycan analysis, HILIC, Ion mobility spectrometry, Calibration, Glucose units, Collision cross sections

## Abstract

LC–MS is one of the most important tools for the comprehensive characterization of *N*-glycans. Despite many efforts to speed up glycan analysis via optimized sample preparation (e.g., faster enzyme digestion in combination with instant or rapid labeling dyes), a major bottleneck remains the rather long measurement times of HILIC chromatography. Further complication arises from the necessity to concomitantly calibrate with an external standard to allow for accurate retention times and the conversion into more robust GU values. Here we demonstrate the use of an internal calibration strategy for HILIC chromatography to speed up glycan analysis. By reducing the number of utilized dextran oligosaccharides, the calibrant can be spiked directly into the sample such that external calibration runs are no longer required. The minimized dextran ladder shows accurate GU calibration with a minor deviation of well below 1% and can be applied without modifications in sample preparation or data processing. We further demonstrate the simultaneous use of the minimized dextran ladder as calibrant for the estimation of CCS values in traveling wave ion mobility spectrometry. In both cases, the minimized dextran ladder enables the measurement of calibrant and sample in a single HPLC run without losing information or accuracy.

**Graphical abstract:**

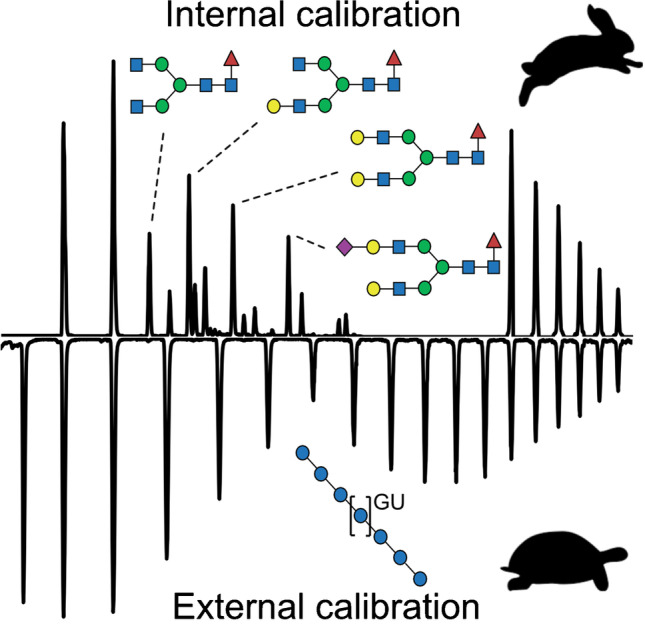

**Supplementary Information:**

The online version contains supplementary material available at 10.1007/s00216-022-04133-0.

## Introduction

Protein glycosylation plays a major role in many biological processes and can affect function, stability, and solubility of the glycoprotein [1]. Due to its importance, monitoring of *N*-glycosylation is a crucial requirement in the characterization of therapeutic antibodies [2] and also gained increased attention to identify potential biomarkers for cancer and other diseases [3–5]. The characterization of *N*-glycans usually requires the cleavage of the glycans from the protein and a subsequent reducing end modification with a fluorescent label [6, 7]. The following analysis is often based on liquid chromatography (LC) in combination with fluorescence (FLD) or mass spectrometry (MS) detection [8, 9]. Although fluorescent labeling is not necessary for MS detection, many labels offer improved ionization properties (such as procainamide in positive ion mode) and therefore represent a popular choice to simplify MS analysis [10]. MS-based sequencing is generally a powerful approach for the analysis of biomolecules, but it is often brought to its limit by the broad variety of glycan isomers. LC separation therefore takes a crucial role in the routine analysis of *N*-glycans. The combination of retention time and *m*/*z* can be stored in databases and is essential for the identification of unknown samples [11]. However, retention times are highly dependent on instrumental parameters and can therefore not be directly used in databases. For this reason, an external calibration with a standard glucose homopolymer—usually referred to as dextran ladder—is indispensable to convert retention times into robust, comparable values, the so-called glucose units (GU) [12, 13]. For larger sets of samples, multiple calibration runs in regular intervals are necessary to achieve accurate GU values. In ideal cases, each sample run is accompanied by a second dextran ladder run to maximize identification confidence. This calibration procedure significantly increases the measurement time and prevents a higher throughput in LC-FLD and LC–MS workflows. New developments for creating relative retention times are therefore necessary to increase the speed of *N*-glycan analysis.

Here, we present a dextran ladder with a reduced number of oligosaccharides as internal calibration standard to reduce absolute measurement time. By minimizing the number of dextran oligosaccharides, the calibrant can be spiked directly into the sample without generating overlapping signals. This allows the qualitative and quantitative analysis of *N*-glycans in a single LC run. We demonstrate the use of the minimized dextran ladder for GU calibration in HILIC chromatography with the released glycans of human immunoglobulin (hIgG) as reference. Furthermore, we illustrate the use of the minimized dextran ladder for the estimation of collision cross-section (CCS) values from traveling wave (TW) ion mobility-mass spectrometry (IM-MS) experiments.

## Materials and methods

### Chemicals

All chemicals and reagents were at least analytical reagent grade and used without further purification. Rapid™ PNGase F and Rapid™ PNGase F Buffer were supplied by New England Biolabs (Ipswich, USA). Immunoglobulin from human serum (hIgG, ≥ 95%), human alpha-1-acid glycoprotein (hAGP, ≥ 95%), dextran Mw5000, dextran Mw1000, Discovery Glycan solid-phase extraction (SPE) tubes, and procainamide hydrochloride (≥ 98%) were purchased from Sigma-Aldrich (St. Louis, USA). Ammonium formate (> 99%) was obtained from VWR International (Radnor, USA). All solvents (acetonitrile, water) were LC–MS grade and purchased from Sigma-Aldrich (St. Louis, USA).

### Sample preparation for labeled glycans

Ten microliters of glycoprotein stock solution (10 mg/mL in water) was mixed with 6 μL water and 4 μL Rapid™ PNGase F Buffer and denatured at 95 °C for 10 min. After cooling to room temperature, 1 μL of Rapid™ PNGase F was added to the mixture and incubated at 50 °C for 10 min. To create the dextran calibration ladder, a 1:1 mix of dextran 5000 and dextran 1000 was dissolved in 20 μL water. Afterwards, both the released glycans and the dextran mix were labeled with procainamide according to established protocols [7, 14]. The labeled glycans were purified with the Discovery Glycan SPE tubes according to vendor’s instruction, dried via SpeedVac (Thermo Fisher Scientific, Waltham, USA) and further suspended in 50 μL water before storing them in the HPLC autosampler at 4 °C.

### Offline IM-MS experiments

Drift tube (DT) IM-MS measurements were performed on a modified Synapt G2-S HDMS instrument (Waters Corporation, Manchester, UK), described in detail elsewhere [15]. Direct infusion measurements were performed in positive ion mode using platinum/palladium (Pt/Pd, 80/20)-coated borosilicate capillaries prepared in-house. For nanoelectrospray ionization (nESI), typically 5 μL of sample was loaded to a capillary and electrosprayed by applying a capillary voltage of 0.6–1.1 kV.

### Online HILIC-IM-MS experiments

Procainamide-labeled glycans were separated by a glycan BEH amide column (150 mm × 2.1 mm, 130 A, 1.7 μm, Waters, Milford, USA) before ESI ionization. Solvent A was 50 mM ammonium formate adjusted to pH 4.4, and solvent B was acetonitrile. The column temperature was set to 65 °C, and samples were analyzed at a flow rate of 0.4 mL/min using a linear gradient of 75–48% B from 0 to 40 min. The injection volume was 4–5 μL. The separated glycans were then ionized online with a capillary voltage of 2.2–2.5 kV.

Typical MS parameters in resolution mode (offline and online measurements) for positive ion polarity were 30 V sampling cone voltage, 1 V source offset voltage, 120 °C source temperature, 0 V trap CE (MS) up to 30 V trap CE (MS/MS), 2 V transfer CE, and 3 mL/min trap gas flow. Ion mobility parameters were 5.0 V trap DC entrance voltage, 5.0 V trap DC bias voltage, − 10.0 V trap DC voltage, 2.0 V trap DC exit voltage, − 25.0 V IMS DC entrance voltage, 50–180 V helium cell DC voltage, − 40.0 V helium exit voltage, 50–150 V IMS bias voltage, 0 V IMS DC exit voltage, 5.0 V transfer DC entrance voltage, 15.0 V transfer DC exit voltage, 150 m/s trap wave velocity, 1.0 V trap wave height voltage, 1000 m/s IMS wave velocity, 40.0 V IMS wave height voltage, 200 m/s transfer wave velocity, and 5.0 V transfer wave height voltage.

Data was acquired with MassLynx v4.1 and processed with Driftscope version 2.8 software (Waters, Manchester, UK), and OriginPro 8.5 (OriginLab Corporation, Northampton).

### CCS determination

Absolute ^DT^CCS_N2_ values were determined via the Stepped-Field method as described elsewhere [16, 17]. In short, each sample was measured at eight different drift voltages with increasing axial potential across the drift tube. For each ion, the corresponding arrival time distribution (ATD) is recorded and extracted by fitting a Gaussian distribution to the raw data. The extrapolation of the reversed axial potential against ATD gives an intercept that is equivalent to the dead time required to transport the ions from the end of the drift tube to the detector. Subtracting the dead time from the observed ATD results in the corrected drift time of each ion required to actually traverse the drift cell. This corrected drift time can be used to calculate the mobility of the ion, which can be inserted into the Mason-Schamp equation to determine CCS values.

Estimated ^TW^CCS_N2_ values were obtained by measuring a calibrant (procainamide-labeled dextran) at a fixed TW height and speed [16]. The drift times were extracted by fitting the ATDs of the ions with a Gaussian distribution and plotted in a logarithmic scale against the corresponding absolute CCS values obtained before. A linear regression can then be used as a calibration curve to estimate CCS. Further details about the calibration procedure can be found in the Supporting Information.

### Fractionation of dextran oligomers

Procainamide-labeled dextran glycans (as described above) were separated by a glycan BEH amide column (150 mm × 2.1 mm, 130 A, 1,7 μm, Waters, Milford, USA) before fluorescence detection (*λ*_ex_ = 310 nm, *λ*_ex_ = 370 nm). Solvent A was 50 mM ammonium formate adjusted to pH 4.4, and solvent B was acetonitrile. The column temperature was set to 65 °C and samples were analyzed at a flow rate of 0.4 mL/min using a linear gradient of 75–48% B from 0 to 40 min. Each dextran oligomer was fractionated manually by observing the fluorescent signal and collected in 1.5-mL tubes. Individual fractions were aliquoted and then mixed to create different combinations of dextran oligomers (see “Results”). The dextran oligomer mixes were then dried via SpeedVac (Thermo Fisher Scientific, Waltham, USA) and subsequently redissolved with the sample solution containing the released and labeled glycans of IgG. The mix of IgG and dextran glycans was then transferred into an HPLC vial before storing them in the HPLC autosampler at 4 °C for LC-IM-MS measurement.

## Results

### External GU calibration

**Fig. 1:**
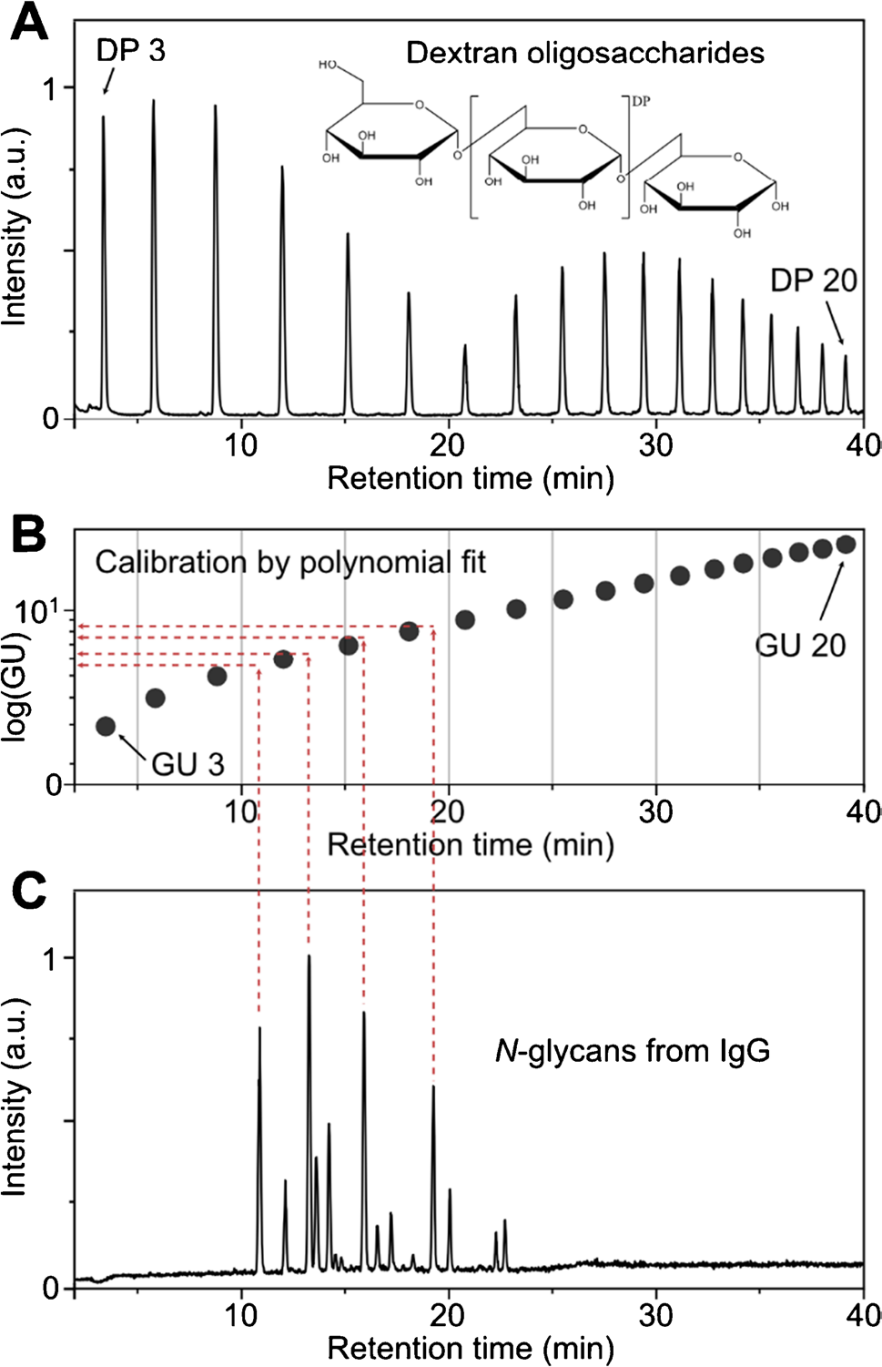
Typical workflow for external GU calibration shown for the released glycans of human IgG. **A** Chromatogram of procainamide-labeled dextran oligomers in HILIC mode with corresponding DP (degree of polymerization) assignment. **B** Plotting GU retention times against log(GU) results in a polynomial shape which can be fitted with a fifth-order equation. **C** Chromatogram of glycans released from human IgG. The resulting polynomial fit of part B can be used for external calibration of unknown samples as indicated by the red-dotted line

The retention behavior of *N*-glycans in HILIC can slightly change over extended periods of time due to external conditions such as column age, alterations in solvent mixture, or system temperature [18, 19]. Although minor drifts are normal, they can impede structural identifications which are based on accurate retention times. External calibration with a homopolymeric glucose standard (dextran ladder) allows for the conversion of retention times into more robust glucose units which can help to minimize this problem. Dextran calibration in HILIC applications is usually performed by plotting a polynomial curve of the respective dextran oligosaccharide retention time against log(GU) (Fig. 1). The polynomial equation of this curve can then be used to calibrate the retention time of unknown samples. The resulting GU values are universally comparable and can therefore be used as references in suitable databases [12, 13, 20]. This is inevitable to simplify structural assignments of unknown glycans.

In order to reach maximum calibration precision, it is necessary to accompany sample runs in regular terms with calibration runs (ideally for each sample) which can double the total measurement time. Coinjection of the calibration standard directly within the sample run is one way to circumvent this increase in measurement time [21, 22]. Although very simple to perform, direct spiking of the complete dextran ladder into the sample leads to overlapping sample and calibrant peaks. LC–MS-based experiments can circumvent this by separating calibrant and sample signals based on exact masses. However, overlapping signals interfere with spectroscopic detection methods such as fluorescence or UV detection, which in turn complicates quantification. As fluorescence detection is commonly employed for glycan analysis, coinjection of calibrant and sample seems impractical without modifications. For applications involving capillary electrophoresis, Guttman et al. circumvent the problem of overlapping peaks by reducing the absolute number of spiked oligosaccharides [23]. Based on only three data points, they generate a virtual calibration ladder to determine GU values for CE, empirically. For HILIC applications, however, no comparable approach for internal calibration was reported although the potential time savings for HPLC runs (hours) would be much larger compared to short CE runs (seconds to minutes).

### Internal GU calibration

**Fig. 2:**
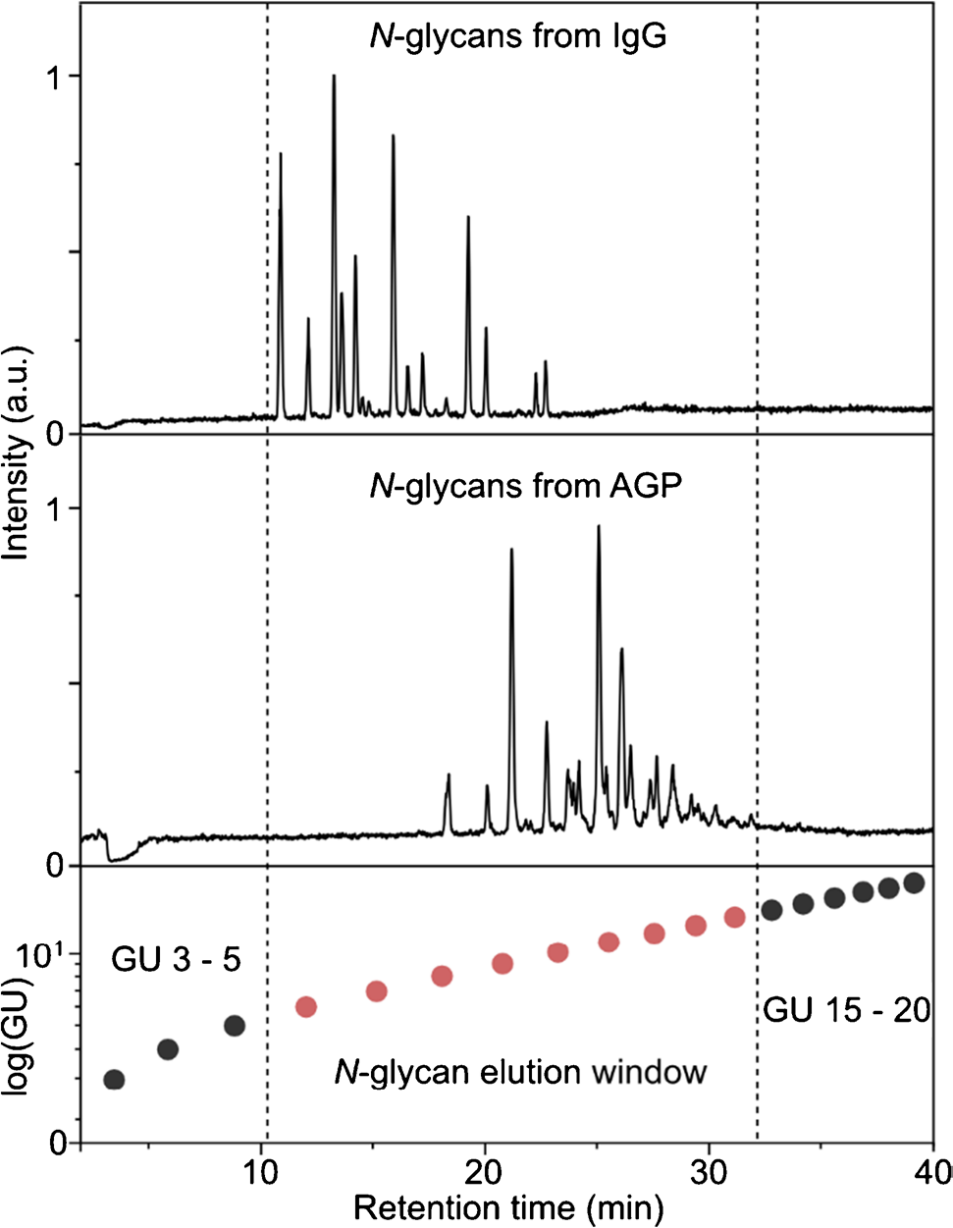
Determination of *N*-glycan elution window for HILIC HPLC. HILIC chromatograms of released glycans of IgG (top panel) and AGP (middle panel). The smallest *N*-glycans from IgG start to elute at ~ 10 min and largest glycans from AGP stop to elute at ~ 32 min (indicated by dashed lines). GU data points inside the *N*-glycan elution window cannot be used for internal calibration, and therefore are marked in red. Internal calibration can make use of GU 3 to 5 and GU 15 to 20

To adapt the dextran ladder for internal calibration in HILIC applications, we have to consider three major points:

1. Typical gradient times in HILIC *N*-glycan analysis are up to 40 min and result in a total run time of 60 min per sample (including washing and re-equilibration steps) [24]. To avoid extension of the original measurement time, only dextran oligosaccharide peaks observed in this gradient window can be considered. The chromatogram of the dextran ladder shows 20 peaks in this time frame. The dextran mono- and disaccharides (GU 1 and 2) usually elute with the injection peak, together with excess label and salts. In the intended internal calibration, these signals therefore have to be excluded and only the GU data points from 3 to 20 can be considered (Fig. 1A + B).
2. The calibrant signals should not overlap with sample signals to allow qualification and relative quantification via spectroscopic detection (e.g., fluorescence or UV). Similar to the CE approach reported by Guttman et al. [23], this makes it necessary to reduce the number of spiked dextran oligosaccharides to omit an elution window in the HILIC chromatogram (Fig. 2). The spiked dextran peaks only should occur before and after the elution window in which the actual sample elutes from the column. To determine the elution window for the applied gradient, the released *N*-glycans of human IgG and human AGP are used as reference points. Human IgG glycosylation mainly consists of complex biantennary *N*-glycans with a high degree of fucosylation but low levels of sialylation [2]. It can therefore serve as lower limit for the *N*-glycan elution window. As an example for larger glycan structures, human AGP can be applied as it mostly contains fully sialylated bi-, tri-, and tetraantennary *N*-glycan structures [25]. A complete identification of all hAGP glycans can be found in the Supporting Information (see Table S-1). With these two sets of samples, it is possible to estimate the elution window for a vast majority of *N*-glycans. Figure 2 shows the HILIC separation of the *N*-glycans from IgG and AGP with the corresponding external GU calibration. The chromatograms reveal that the smallest *N*-glycans from human IgG start to elute after GU 5 while the largest *N*-glycans from AGP are eluted before GU 15. This means that typical *N*-glycans are released from the columns within an elution window of ~ 10 to ~ 32 min or between GU values 5 and 15, respectively. To avoid overlapping signals of sample and calibrant, it is therefore necessary to omit the GU values between 6 and 14. An internal dextran calibration should hence only rely on the GU values 3 to 5 and 15 to 20.
3. The most important part is the quality of the calibration. The internal dextran calibration needs to be as accurate as the routinely used external calibration. For this purpose, different combinations of dextran oligosaccharides of GU 3 to 5 and 15 to 20 were evaluated (see Figure S-2 in the Supporting Information). Although the absolute number of data points is reduced (especially in the *N*-glycan elution window), the polynomial fit has to compensate for this to resemble the actual GU values. The combination of GU 4 + 5 and GU 15 to 20 resulted in the most accurate polynomial fit and nicely resembles the external calibration (see Table S-3 and Table S-4 in the Supporting Information). This combination of data points is therefore utilized as internal dextran ladder for the following experiments.

**Fig. 3:**
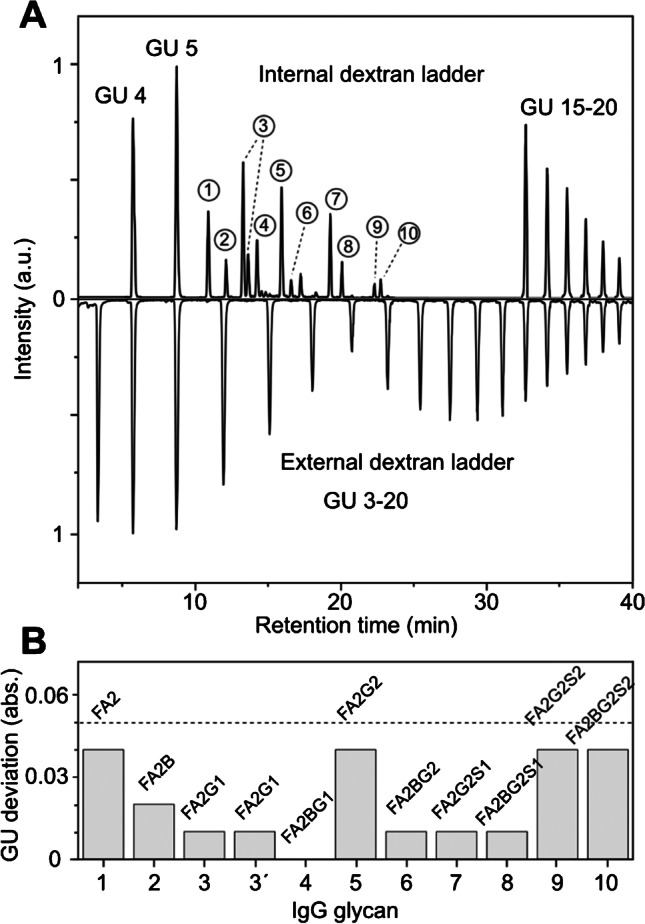
Evaluation of the internal GU calibration on the example of human IgG. **A** Mirror plot of HILIC chromatograms of released *N*-glycans from IgG spiked with the minimized dextran ladder compared to the complete external dextran ladder. The numbers in circles indicate the identified *N*-glycan species from IgG. **B** Bar graph of GU deviation (in absolute numbers) between internal and external calibration. The dashed line indicates the GU tolerance typically applied in database searches

To generate the minimized set of standards, all relevant dextran oligosaccharides were fractionated, and then, a small aliquot was spiked into the released glycans of human IgG. Figure 3 shows the HILIC chromatogram with internal dextran calibration. As intended, there is a clear separation of sample (from minutes ~ 10 to ~ 25) and calibrant signals. This allows for the simple discrimination of signals from human IgG and leads to the identification of 11 N-glycans (Fig. 3A, marked with a number). The comparison with the external calibration (mirror plot, Fig. 3) shows that multiple IgG signals overlap with the dextran ladder and would therefore be difficult to quantify based on optical detection methods. Both the internal and the external dextran ladders are used to calculate the GU units of the 11 IgG peaks, and the resulting values are compared in Fig. 3B. Although the minimized dextran ladder uses nine data points less than the full set of standards, the differences between both calibration strategies are surprisingly small (see Table S-5 in the Supporting Information). The highest deviation is 0.04 GU, while most other species show deviations below 0.01 GU. Databases usually apply a GU tolerance of 0.05 to 0.2 [12, 26], which is easily accessible with the internal calibration as indicated by the dashed line in Fig. 3B. As the GU values for procainamide-labeled *N*-glycans of human IgG can be found in the GlycoStore database [20], we further compared the results of the internal and external dextran calibration with GU values of the database (see Table S-6 in the Supporting Information). The GU datasets of both, internal and external, calibrations are in good agreement with the database entries and exhibit average deviations below 1.5%. With these results, we can conclude that the spiked dextran ladder produces an accurate calibration on equal grounds with the external dextran ladder. Spiking of the calibrant into the sample can be easily applied to a large number of samples and can therefore help to speed up the time-consuming calibration procedure.

### CCS calibration

Besides the widespread LC methods for *N*-glycan characterization, IMS emerged as promising orthogonal tool to improve isomer separation [27, 28]. IMS can separate glycan isomers based on their drift times in the gas phase to identify specific glycan motifs [29–31]. The generated drift time behaves similar to retention times of LC applications and represents an instrument-dependent parameter. This means that drift times are affected by external conditions and therefore hard to compare between different instruments (or in databases). This can be avoided by converting the drift times into collision cross sections (CCSs) which describe a molecular property of the ion and are independent of instrumental parameters [32]. For linear drift cells (DTIMS), drift times can be directly converted mathematically using the Mason-Schamp Equation. [33]. Most of the state-of-the-art IMS instruments, however, utilize non-homogeneous electric fields which preclude the determination of absolute CCSs (e.g., TWIMS, TIMS) [8]. To enable the estimation of CCSs, these IMS instruments require careful calibration with a suitable standard. The calibrant should ideally be of the same biomolecular class and in the same *m*/*z* range as the actual sample. For CCS calibration of glycans, dextran is regularly used as external standard as it is well-characterized and readily available [16, 29].

**Fig. 4:**
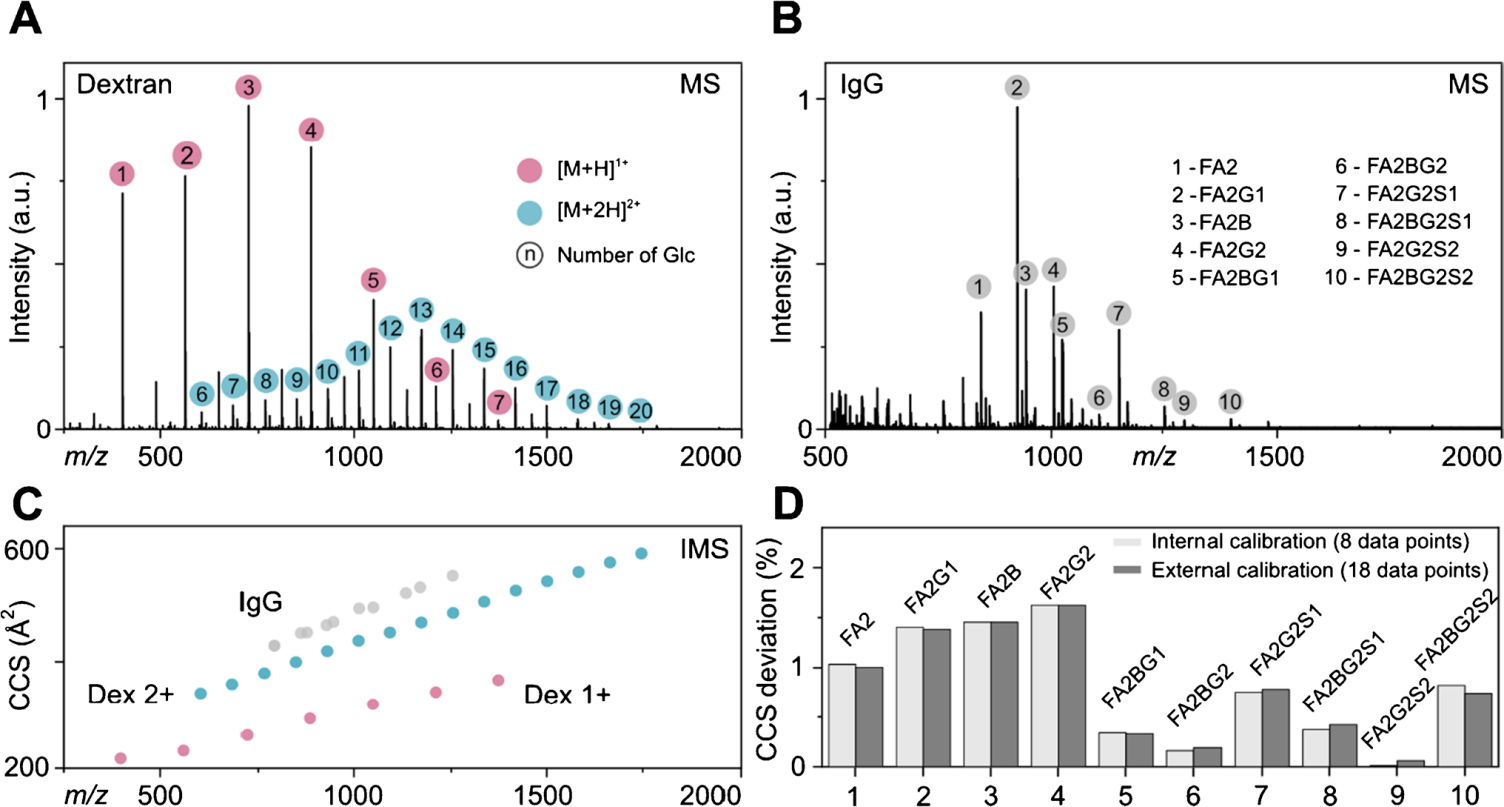
CCS calculation with internal and external dextran ladder. **A** MS spectrum of procainamide-labeled complete dextran ladder measured by direct injection IM-MS. Singly charged species are labeled with a red circle while doubly charged species are highlighted in blue. **B** MS spectrum of procainamide-labeled *N*-glycans of human IgG measured by direct injection IM-MS. **C** CCS plot of absolute.^DT^CCS_N2_ values of dextran and IgG measured by direct injection IM-MS. **D** Bar chart of CCS deviation for internal (only drift times of minimized dextran ladder were utilized, 8 data points) and external calibration (drift times of all dextran oligomers were utilized, 18 data points) measured by LC-IM-MS in comparison to absolute CCS values

The IMS calibration process requires absolute ^DT^CCS values as reference. We therefore measured offline IM-MS data for the procainamide-labeled glycans of dextran and IgG on a modified Synapt G2 instrument equipped with a linear drift cell (Fig. 4). The resulting mass spectra of dextran glycans show oligomers starting from monomers up to 20-mers with charge states ranging from 1 + to 2 + (Fig. 4A). This covers basically all types of *N*-glycans as the typical *m*/*z* range starts from biantennary glycans (*m*/*z* 500–1000 with charge states 1 +) and goes up to fully sialylated tetraantennary glycans (*m*/*z* up to 4000 with charge states 2 + /3 +). The typical mass range for released glycans from human IgG are presented in Fig. 4B and shows doubly charged species ranging from *m*/*z* ~ 750 to 1250. For all these species, absolute ^DT^CCS_N2_ were determined (Fig. 4C). The singly charged oligosaccharides of dextran cover a CCS range from ~ 200 to ~ 400 Å^2^, while the doubly charged dextran oligomers cover a bigger span of ~ 300 to ~ 600 Å^2^. The glycans of IgG are inside this limit and range from ~ 400 to ~ 550 Å^2^. As dextran covers the complete mass and CCS range, it is suitable as calibrant for IgG. To evaluate the accuracy of the CCS calibration with either the complete dextran ladder or the reduced dextran ladder, we ran three individual HILIC runs. We measured the complete dextran ladder and the released glycans of human IgG as individual samples, and in the third run, a measurement of the reduced dextran ladder directly spiked to the IgG glycans was performed. The drift times obtained from the LC-IM-MS runs were then used for either external or internal CCS calibration. For both, complete and reduced, dextran ladders, we observe singly protonated species for GU 3 to 11 and the doubly protonated species for GU 8 to 20. In both cases, we only utilized the drift times of species that have a reference value measured by offline IM-MS (see Table S-7). External calibration was performed with the complete dextran ladder (18 data points including GU 3–7 as singly charged species (Figure S-8) and GU 8–20 as doubly charged species (Figures S-9 and S-10)). Internal CCS calibration only utilized drift times corresponding to the reduced dextran ladder (8 data points including GU 4 + 5 as singly charged ions and GU 15–20 as doubly charged ions). The calibration curves created by the internal and external dextran ladders were then used to estimate the ^TW^CCS_N2_ for the IgG glycans. For a comprehensive table of all calculated CCS values, see Table S-11. To evaluate the estimated ^TW^CCS_N2_, we compared both datasets (external and internal calibration) to the calculated absolute ^DT^CCS_N2_ values from IgG (Fig. 4D). The CCS deviation for each glycan is shown as bar plot to visualize the negligible differences between internal and external calibration (< 0.1%). Both datasets follow a similar trend with decreasing CCS deviation for increasing *m*/*z* ratio. The error is highest for the smallest glycans (FA2 to FA2G2) with 1 to 1.5% and decreases for the largest glycans to well below 1%. The average CCS deviation over all glycans is < 0.8% and therefore in the order of the general calibration error reported for CCS estimations [17, 34]. Similar to the GU calibration discussed above, CCS calibration with a reduced number of dextran oligosaccharides does not reduce the accuracy of the calibration. The internal calibration therefore results in very similar GU and CCS values compared to the external calibration, but has a significant advantage with respect to throughput.

## Conclusion

In this study, we established an internal calibration approach for the analysis of released *N*-glycans via LC in conjunction with FLD and/or IM-MS. For the internal calibration, we reduced the number of dextran oligomers to spike the dextran ladder directly into the sample. It was found that 8 dextran oligosaccharides are sufficient for accurate calibration. When used for GU calibration, the minimized dextran ladder can be clearly discriminated from sample signals in the HILIC chromatogram and therefore does not disturb quantification via optical detection. Although the absolute number of data points for calibration is reduced, the minimized dextran ladder exhibits negligible deviations from the external GU calibration and allows for accurate determination of GU glucose units. The situation is very similar for CCS calibration. The reduced number of dextran oligosaccharides did not lead to a loss in calibration accuracy, and only minor differences to the external calibration are observed. Furthermore, the GU and CCS datasets generally exhibit very low error margins and therefore are readily compatible to established databases such as GlycoStore or GlycoMob [20, 35].

The proposed internal calibration approach has the largest potential for LC-FLD and LC-FLD-IM-MS applications, as it not only simplifies the GU annotation of *N*-glycans, but also enables a straightforward relative quantification based on the fluorescence signal. However, also MS- and IMS-based workflows without FLD detection can benefit from internal calibration. In comparison to external calibration, which requires the same running conditions for all sample runs, the proposed internal calibration offers much more flexibility. Each LC-IM-MS run could be adapted in terms of instrumental conditions (resolution, ion accumulation) or based on specific sample properties in case of stability issues. Most modern IMS instrument are tunable in terms of IMS cycles (cyclic IMS, SLIMS) or voltage ramps (TIMS) and could therefore make use of the flexibility offered by internal calibration. For large sample sets, the internal dextran ladder can further be used to assess and, if necessary, re-calibrate IMS measurements. Similar to lock masses in MS calibration, the internal dextran ladder can provide an ongoing calibration to compensate for gas and temperature fluctuations when measuring over extended periods of time.

For the internal calibration, dextran proved to be a universal standard for the analysis of *N*-glycans. It is readily available, cheap, and well-characterized. Once the dextran oligosaccharides are fractionated, the direct spiking of calibrant into the sample significantly reduces the measurement time as there is no need for additional external LC or CCS calibration runs. It further requires minimal adaptations in data acquisition and processing as the general calibration procedures does not change. It can therefore directly replace external calibration to save time and increase the throughput in *N*-glycan analysis.

## Supplementary Information

Below is the link to the electronic supplementary material.

Supplementary file1 (DOCX 475 KB)
