# Supplementary material for: Dextran as internal calibrant for N-glycan analysis by liquid chromatography coupled to ion mobility-mass spectrometry
Source: Anal Bioanal Chem. 2022 May 26;414(17):5023–31. doi: 10.1007/s00216-022-04133-0 (PMC9234027; doi:10.1007/s00216-022-04133-0)
Supplement: Supplementary file 1 — Supplementary file1 (DOCX 475 KB) [file 216_2022_4133_MOESM1_ESM.docx]

**Supporting Information for**

**Dextran as Internal Calibrant for *N*-Glycan Analysis by Liquid Chromatography Coupled to Ion Mobility-Mass Spectrometry**

Christian Manz, Michael Götze, Clemens Frank, Andreas Zappe, Kevin Pagel*

Freie Universität Berlin, Department of Chemistry and Biochemistry, Arnimallee 22, 14195 Berlin, Germany

Fritz Haber Institute of the Max Planck Society, Department of Molecular Physics, Faradayweg 4-6, 14195 Berlin, Germany

* Corresponding author. E-Mail: kevin.pagel@fu-berlin.de

**Table S-1**: **Compositional analysis of released and procainamide-labeled *N*-glycans from human AGP based on HILIC LC-MS.**

| **Retention time** | **Species** | ***m/z*** | **z** | **m** | **Hex** | **HexNAc** | **Fuc** | **Sia** |
| --- | --- | --- | --- | --- | --- | --- | --- | --- |
| 17.4 | A2G2S1 | 1076.43 | 2 | 2150.86 | 5 | 4 | 0 | 1 |
| 17.6 | A2G2S1 | 1076.43 | 2 | 2150.86 | 5 | 4 | 0 | 1 |
| 18.6 | A2G2S1 | 1076.43 | 2 | 2150.86 | 5 | 4 | 0 | 1 |
| 18.7 | A2G2S1 | 1076.43 | 2 | 2150.86 | 5 | 4 | 0 | 1 |
| 20.3 | FA2G2S2 | 1294.99 | 2 | 2587.98 | 5 | 4 | 1 | 2 |
| 21.3 | FA2G2S2 | 1294.99 | 2 | 2587.98 | 5 | 4 | 1 | 2 |
| 22.3 | FA2G2S2 | 1294.99 | 2 | 2587.98 | 5 | 4 | 1 | 2 |
| 22.5 | FA2G2S2 | 1294.99 | 2 | 2587.98 | 5 | 4 | 1 | 2 |
| 20.6 | A2G2S2 | 1221.98 | 2 | 2441.96 | 5 | 4 | 0 | 2 |
| 21.7 | A2G2S2 | 1221.98 | 2 | 2441.96 | 5 | 4 | 0 | 2 |
| 22.2 | A3G3S2 | 1404.54 | 2 | 2807.08 | 6 | 5 | 0 | 2 |
| 23.2 | A3G3S2 | 1404.54 | 2 | 2807.08 | 6 | 5 | 0 | 2 |
| 24.1 | A3G3S2 | 1404.54 | 2 | 2807.08 | 6 | 5 | 0 | 2 |
| 24.7 | A3G3S3 | 1550.07 | 2 | 3098.14 | 6 | 5 | 0 | 3 |
| 25.6 | A3G3S3 | 1550.07 | 2 | 3098.14 | 6 | 5 | 0 | 3 |
| 26.5 | A3G3S3 | 1550.07 | 2 | 3098.14 | 6 | 5 | 0 | 3 |
| 25.7 | FA3G3S3 | 1623.08 | 2 | 3244.16 | 6 | 5 | 1 | 3 |
| 26.2 | FA3G3S3 | 1623.08 | 2 | 3244.16 | 6 | 5 | 1 | 3 |
| 26.6 | FA3G3S3 | 1623.08 | 2 | 3244.16 | 6 | 5 | 1 | 3 |
| 26.1 | A4G4S3 | 1732.60 | 2 | 3463.2 | 7 | 6 | 0 | 3 |
| 27.0 | A4G4S3 | 1732.60 | 2 | 3463.2 | 7 | 6 | 0 | 3 |
| 27.6 | A4G4S3 | 1732.60 | 2 | 3463.2 | 7 | 6 | 0 | 3 |
| 28.2 | A4G4S4 | 1878.12 | 2 | 3754.24 | 7 | 6 | 0 | 4 |
| 28.9 | A4G4S4 | 1878.12 | 2 | 3754.24 | 7 | 6 | 0 | 4 |
| 28.9 | FA4G4S4 | 1951.11 | 2 | 3900.22 | 7 | 6 | 1 | 4 |
| 29.3 | FA4G4S4 | 1951.11 | 2 | 3900.22 | 7 | 6 | 1 | 4 |
| 29.7 | FA4G4S4 | 1951.11 | 2 | 3900.22 | 7 | 6 | 1 | 4 |


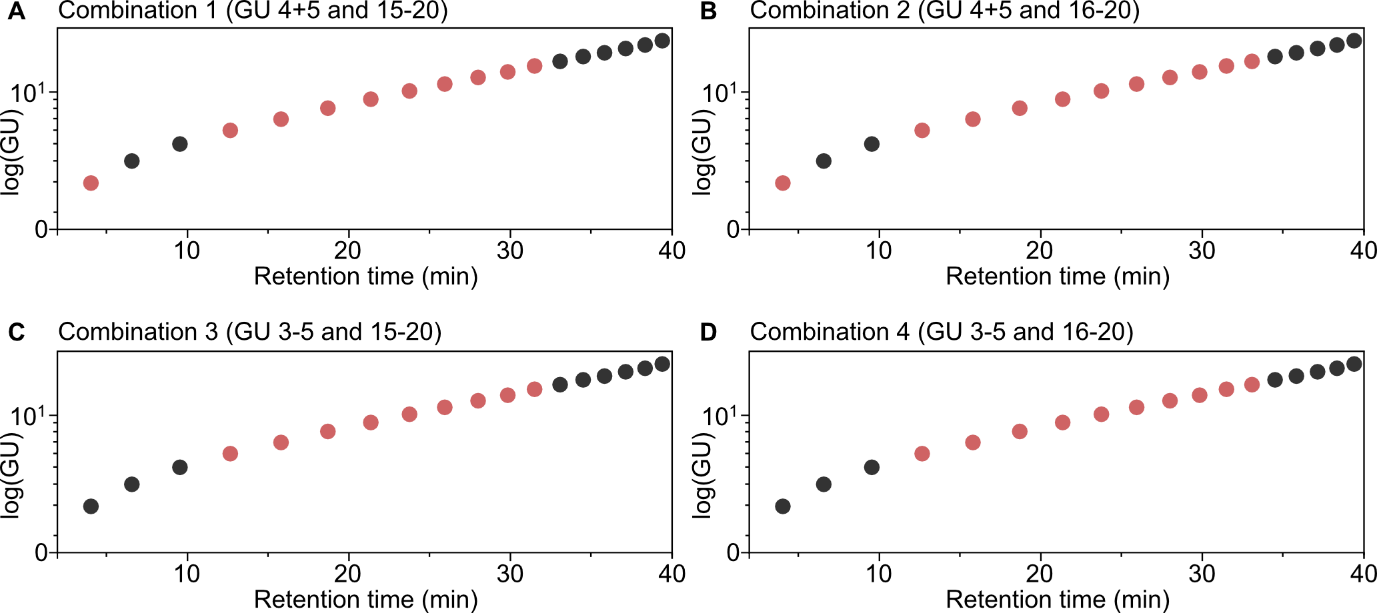


**Figure S-2: Generation of polynomial fits for different combinations of minimized dextran ladder.** GU 1 and GU 2 (dextran mono- and disaccharides) are ignored in all cases, as they elute early together with access label and salts and therefore are impractical to use for accurate calibration curves. As described in the main manuscript, GU 6-14 are also left out to leave an *N*-glycan elution window. Only GU 3-5 and 15-20 are therefore considered for an accurate fit. To determine the accuracy of the respective fit, a polynom of fifth grade only based on the reduced number of data points was generated (individual for each combination) and used to generate absolte GU values (see table S-2). (a) Combination 1 utilizes 8 data points (GU 4+5 and 15-20). (b) Combination 2 utilizes 7 data points (GU 4+5 and 16-20). (c) Combination 3 utilizes 9 data points (GU 3-5 and 15-20). and (d) Combination 4 utilizes 8 data points (GU 3-5 and 16-20).

**Table S-3: Parameters of polynomial fit for the different combinations of the minimized dextran ladder.** For all tested combinations of the reduced/minimized dextran ladder (1-4) and for the full dextran ladder the parameter for the polynomial equation are given.

| **Fits** | **y = y0 + B1 * x + B2 * x^2 + B3 * x^3 + B4 * x^4 + B5 * x^5** | | | | |
| --- | --- | --- | --- | --- | --- |
| **Parameter** | **all values** | **combination1** | **combination2** | **combination3** | **combination4** |
| **y0** | 0.90290 | 0.93122 | -1.00241 | 0.65512 | 0.65705 |
| **B1** | 0.65734 | 0.66494 | 1.34183 | 0.76201 | 0.76048 |
| **B2** | -0.03527 | -0.03714 | -0.11730 | -0.04873 | -0.04835 |
| **B3** | 0.00161 | 0.00168 | 0.00560 | 0.00226 | 0.00222 |
| **B4** | -0.00003 | -0.00003 | -0.00012 | -0.00005 | -0.00004 |
| **B5** | 0.00000 | 0.00000 | 0.00000 | 0.00000 | 0.00000 |

**Table S-4: Calculated GU values for the different combinations of the minimized dextran ladder.** GU values are calculated based on the polynomial fit generated in figure S-1. The theoretical GU values serve as reference to evaluate the calculated GU values for the different combinations. The GU values in the range of 6-14 are highlighted as the majority of *N*-glycans should elute in this time frame. The calculated GU values in this range should therefore be as accurate as possible to calibrate for actual samples. Combination 1 shows the highest accuracy in this range (deviations <0.09) and exhibits very similar values to the theoretical GU values and the external GU calibration which utlizes all data points.Combinations 2-4 show larger deviations >> 0.1 GU, especially in the smaller GU range of 6-11. Therefore less data points than the ones used in combination 1 seem impractical and were not further tested.

| **GU-Units** | | **all values** | **combi 1** | **combi2** | **combi 3** | **combi 4** |
| --- | --- | --- | --- | --- | --- | --- |
| **RT** | **GU** |  |  |  |  |  |
| 3.88 | 3 | 3.01 | 3.04 | 2.74 | 3.00 | 3.00 |
| 6.25 | 4 | 3.98 | 4.00 | 4.00 | 4.00 | 4.00 |
| 9.2 | 5 | 5.01 | 5.00 | 5.00 | 5.00 | 5.00 |
| 12.37 | **6** | **6.01** | **5.98** | **5.78** | **5.95** | **5.95** |
| 15.49 | **7** | **7.00** | **6.94** | **6.56** | **6.89** | **6.89** |
| 18.39 | **8** | **7.99** | **7.92** | **7.45** | **7.86** | **7.85** |
| 21.05 | **9** | **8.99** | **8.91** | **8.45** | **8.85** | **8.84** |
| 23.48 | **10** | **10.00** | **9.92** | **9.53** | **9.87** | **9.86** |
| 25.69 | **11** | **11.00** | **10.94** | **10.64** | **10.90** | **10.89** |
| 27.71 | **12** | **12.01** | **11.96** | **11.76** | **11.94** | **11.92** |
| 29.55 | **13** | **13.00** | **12.97** | **12.85** | **12.96** | **12.95** |
| 31.26 | **14** | **14.01** | **13.99** | **13.93** | **13.98** | **13.98** |
| 32.83 | 15 | 15.00 | 15.00 | 14.98 | 15.00 | 14.99 |
| 34.28 | 16 | 16.00 | 16.00 | 16.00 | 16.00 | 16.00 |
| 35.62 | 17 | 16.99 | 17.00 | 17.00 | 17.00 | 17.00 |
| 36.88 | 18 | 17.99 | 18.00 | 18.00 | 18.00 | 18.00 |
| 38.06 | 19 | 19.00 | 19.00 | 19.00 | 19.00 | 19.00 |
| 39.16 | 20 | 20.01 | 20.00 | 20.00 | 20.00 | 20.00 |

**Table S-5: GU deviation between external and internal dextran calibration for the released glycans of IgG.** The differences in absolute GU values (between external and internal calibration) and the difference relative to the GU value from the external calibration are broken down for each glycan species of human IgG.

| **LC peak no.** | **Glycan** | **GU int** | **GU  ext** | **Deviation (abs.)** | **Deviation  (in %)** |
| --- | --- | --- | --- | --- | --- |
| 1 | FA2 | 5.66 | 5.70 | 0.04 | 0.70 |
| 2 | FA2B | 6.05 | 6.07 | 0.02 | 0.33 |
| 3 | FA2G1 | 6.41 | 6.42 | 0.01 | 0.16 |
| 3 | FA2G1 | 6.52 | 6.53 | 0.01 | 0.15 |
| 4 | FA2BG1 | 6.71 | 6.71 | 0.00 | 0.00 |
| 5 | FA2G2 | 7.21 | 7.25 | 0.04 | 0.55 |
| 6 | FA2BG2 | 7.47 | 7.46 | 0.01 | 0.13 |
| 7 | FA2G2S1 | 8.28 | 8.29 | 0.01 | 0.12 |
| 8 | FA2BG2S1 | 8.41 | 8.40 | 0.01 | 0.12 |
| 9 | FA2G2S2 | 9.55 | 9.59 | 0.04 | 0.42 |
| 10 | FA2BG2S2 | 9.73 | 9.77 | 0.04 | 0.41 |
|  |  |  | **Avg. deviation** | **0.02 GU** | **0.28%** |

**Table S-6: Comparison of calculated internal and external GU values of IgG with database values.** The calculated values of the internal dextran ladder (8 data points) is very good agreement with reference GU values from glycostore. The biggest deviation is 0.21 GU for FA2BG2S1, while the average deviations is around 0.106 GU. The errror is therefore well below 1.5% for all identified glycans from IgG. A similar picture can be seen for the external calibration. Although the largest deviation is a little bit bigger with 0.23 GU for the largest glycan FA2BG2S2, the general trends for deviations are very similar and also the average deviation is roughly the same with 0.114 GU.

| **peak no.** | **Glycan** | **Ref**  **.** | **GU int** | **dev**  **(abs)** | **dev**  **(%)** | **GU**  **ex** | **dev**  **(abs)** | **dev**  **(%)** |
| --- | --- | --- | --- | --- | --- | --- | --- | --- |
| 1 | FA2 | 5.66 | 5.66 | 0.00 | 0.00 | 5.70 | 0.04 | 0.71 |
| 2 | FA2B | 6.06 | 6.05 | 0.01 | 0.17 | 6.07 | 0.01 | 0.17 |
| 3 | FA2G1 | 6.48 | 6.41 | 0.07 | 1.08 | 6.42 | 0.06 | 0.93 |
| 3 | FA2G1 | 6.60 | 6.52 | 0.08 | 1.21 | 6.53 | 0.07 | 1.06 |
| 4 | FA2BG1 | n.A. | 6.71 | n.A. | n.A. | 6.71 | n.A. | n.A. |
| 5 | FA2G2 | 7.35 | 7.21 | 0.14 | 1.90 | 7.25 | 0.10 | 1.36 |
| 6 | FA2BG2 | 7.56 | 7.47 | 0.09 | 1.19 | 7.46 | 0.10 | 1.32 |
| 7 | FA2G2S1 | 8.39 | 8.28 | 0.11 | 1.31 | 8.29 | 0.10 | 1.19 |
| 8 | FA2BG2S1 | 8.62 | 8.41 | 0.21 | 2.44 | 8.40 | 0.22 | 2.55 |
| 9 | FA2G2S2 | 9.39 | 9.55 | 0.16 | 1.70 | 9.59 | 0.20 | 2.13 |
| 10 | FA2BG2S2 | 9.54 | 9.73 | 0.19 | 1.99 | 9.77 | 0.23 | 2.41 |
|  |  | **Avg.** | **dev.** | **0.11 GU** | **1.30%** |  | **0.11 GU** | **1.38%** |

**Table S-7: Absolute CCS values of the dextran ladder obtained by direct infusion IM-MS.** All CCS values are measured in nitrogen in positive ion mode for the singly protonated (1+) or doubly protonated (2+) species. The singly charged species of dextran can be seen up to the 7-mer, while the doubly charged species almost cover a range from GU 3 to GU 20. This list of absolute ^DT^CCS_N2_ is used to generate the calibration curve for estimating ^TW^CCS_N2_ values.

| **Glycan** | **m/z** | **z** | **m** | **CCS (in Å^2^)** |
| --- | --- | --- | --- | --- |
| Dextran GU 1 | 400.24 | 1 | 400.24 | 212.64 |
| Dextran GU 2 | 562.29 | 1 | 562.30 | 228.05 |
| Dextran GU 3 | 724.34 | 1 | 724.35 | 256.11 |
| Dextran GU 4 | 886.39 | 1 | 886.40 | 283.82 |
| Dextran GU 5 | 1048.45 | 1 | 1048.45 | 308.92 |
| Dextran GU 6 | 1210.50 | 1 | 1210.50 | 329.58 |
| Dextran GU 7 | 1372.56 | 1 | 1372.56 | 352.46 |
| Dextran GU 3 | 362.67 | 2 | 724.35 | 325.01 |
| Dextran GU 4 | 443.70 | 2 | 886.40 | 327.72 |
| Dextran GU 5 | 524.72 | 2 | 1048.45 | 331.75 |
| Dextran GU 6 | 605.75 | 2 | 1210.50 | 354.02 |
| Dextran GU 7 | 686.78 | 2 | 1372.56 | 373.15 |
| Dextran GU 8 | 767.80 | 2 | 1534,61 | 392.75 |
| Dextran GU 9 | 848.83 | 2 | 1696.66 | 411.96 |
| Dextran GU 10 | 929.85 | 2 | 1858.72 | 430.14 |
| Dextran GU 11 | 1010.88 | 2 | 2020.77 | 444.69 |
| Dextran GU 12 | 1091.91 | 2 | 2182.82 | 461.99 |
| Dextran GU 13 | 1172.93 | 2 | 2344.87 | 478.73 |
| Dextran GU 14 | 1253.96 | 2 | 2506.93 | 498.37 |
| Dextran GU 15 | 1334.99 | 2 | 2668.98 | 517.84 |
| Dextran GU 16 | 1416.02 | 2 | 2831.03 | 534.17 |
| Dextran GU 17 | 1497.04 | 2 | 2993.08 | 550.28 |
| Dextran GU 18 | 1578.07 | 2 | 3155.14 | 566.94 |
| Dextran GU 19 | 1659.10 | 2 | 3317.19 | 582.57 |
| Dextran GU 20 | 1740.12 | 2 | 3479.24 | 593.90 |

**Figure S-8: Arrival time distributions from direct injection measurements for singly protonated dextran species (listed in table S-7).** Each species was measured *via* stepped-field method with eight different voltages to obtain absolute CCS values with nitrogen as drift gas. Here, only one exemplary ATD is shown for each species for the drift voltage of 172 V (He cell DC: 100 V, He exit: -40 V, Bias: 120 V, Trans entrance: 5 V).


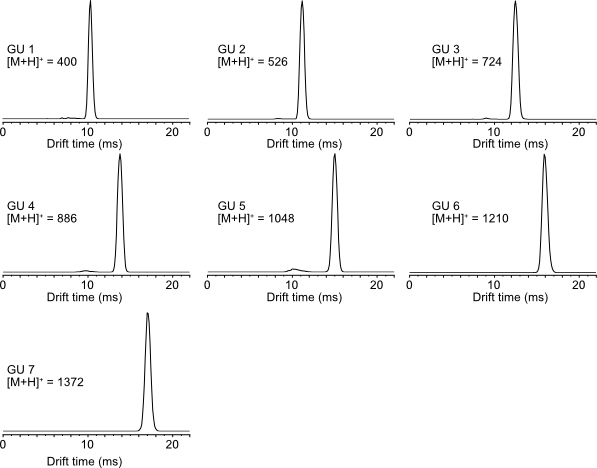


**Figure S-9: Arrival time distributions from direct injection measurements for doubly protonated dextran species from GU 3 to GU 11 (listed in table S-7).** Each species was measured *via* stepped-field method with eight different voltages to obtain absolute CCS values with nitrogen as drift gas. Here, only one exemplary ATD is shown for each species for the drift voltage of 172 V (He cell DC: 100 V, He exit: -40 V, Bias: 120 V, Trans entrance: 5 V).


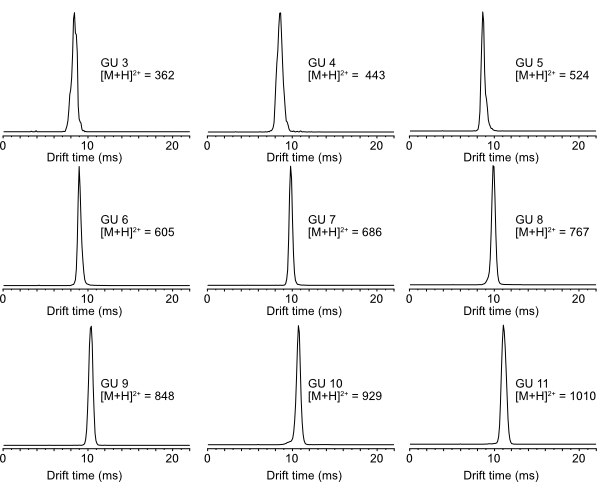


**Figure S-10: Arrival time distributions from direct injection measurements for doubly protonated dextran species from GU 12 to GU 20 (listed in table S-7).** Each species was measured *via* stepped-field method with eight different voltages to obtain absolute CCS values with nitrogen as drift gas. Here, only one exemplary ATD is shown for each species for the drift voltage of 172 V (He cell DC: 100 V, He exit: -40 V, Bias: 120 V, Trans entrance: 5 V).


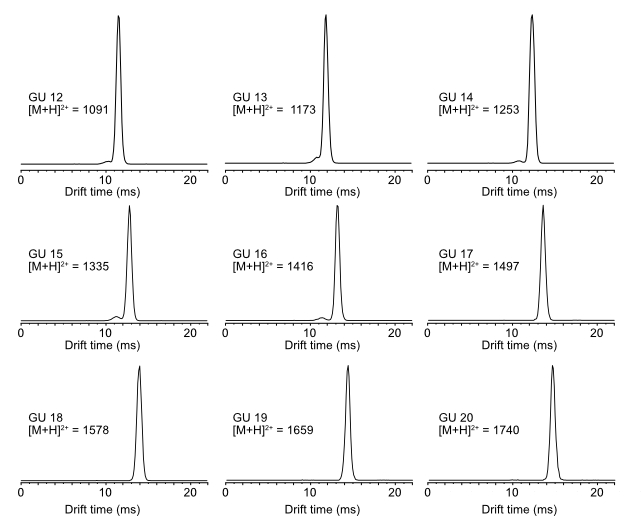


**Table S-11: Comparison of calculated and theoretical CCS from human IgG.** All CCS values are given in Å^2^ and measured in nitrogen in positive ion mode as doubly protonated (2+) species. The absolute ^DT^CCS_N2_ of IgG were measured via direct injection IM-MS and are used here to evaluate the accuracy of the external and internal calibration. The estimated ^TW^CCS_N2_ are generated via a calibration excel sheet^[[1]](#footnote-1)^. In short, the absolute CCS from dextran (table S-6) and the measured TWIMS drift times of each dextran oligosaccharide are used to generate a calibration curve. By using the TWIMS drift time of the IgG glycan, the CCS of the sample can be estimated with help of the calibration curve. All TWIMS drift times were measured with an IMS wave velocity of 1000 m/s and an IMS wave height voltage of 40V.

| **Glycan** | **MS**  **peak no.** | **m/z** | **z** | **^DT^CCS_N2_** | **^TW^CCS_N2_**  **int** | **Dev (in %)** | **^TW^CCS_N2_**  **ext** | **Dev (in %)** |
| --- | --- | --- | --- | --- | --- | --- | --- | --- |
| FA2 | 1 | 841.89 | 2 | 424.56 | 420.16 | 1.04 | 420.33 | 1.00 |
| FA2B | 3 | 943.43 | 2 | 448.41 | 454.90 | 1.45 | 454.92 | 1.45 |
| FA2G1 | 2 | 922.92 | 2 | 446.58 | 440.34 | 1.40 | 440.43 | 1.38 |
| FA2G2 | 4 | 1003.94 | 2 | 467.16 | 468.73 | 0.34 | 468.69 | 0.33 |
| FA2BG1 | 5 | 1024.46 | 2 | 461.20 | 453.73 | 1.62 | 453.76 | 1.61 |
| FA2BG2 | 6 | 1105.48 | 2 | 491.71 | 490.91 | 0.16 | 490.76 | 0.19 |
| FA2G2S1 | 7 | 1149.49 | 2 | 479.72 | 481.5 | 0.37 | 481.4 | 0.35 |
| FA2BG2S1 | 8 | 1251.03 | 2 | 494.34 | 490.61 | 0.75 | 490.47 | 0.78 |
| FA2G2S2 | 9 | 1295.04 | 2 | 520.13 | 518.21 | 0.37 | 517.93 | 0.42 |
| FA2BG2S2 | 10 | 1396.58 | 2 | 530.22 | 530.26 | 0.01 | 529.92 | 0.06 |
|  |  |  |  |  | **Average dev.** | **0.75%** |  | **0.76%** |

**Figure S-12: Arrival time distributions from direct injection measurements for doubly protonated glycans released from human IgG (listed in table S-11).** Each species was measured *via* stepped-field method with eight different voltages to obtain absolute CCS values with nitrogen as drift gas. Here, only one exemplary ATD is shown for each species for the drift voltage of 172 V (He cell DC: 100 V, He exit: -40 V, Bias: 120 V, Trans entrance: 5 V).


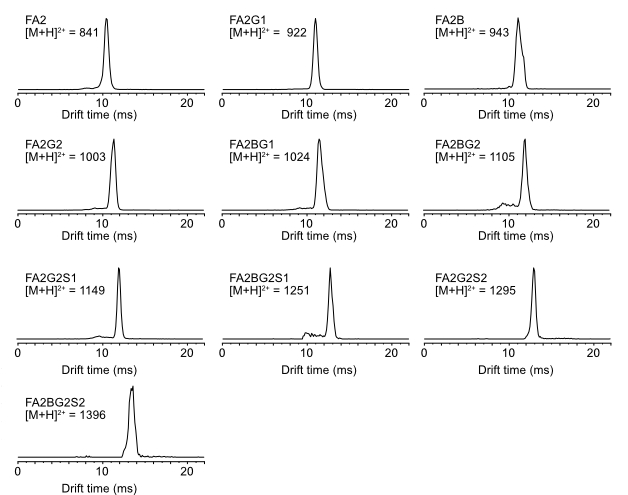


1. The calibration process in general and the utilized excel spreadsheet can be found here: <https://www.bcp.fu-berlin.de/en/chemie/chemie/forschung/OrgChem/pagel/research/carbohydrates/index.html>.

   For further information see: Hofmann, J.; Struwe, W. B.; Scarff, C. A.; Scrivens, J. H.; Harvey, D. J.; Pagel, K. Estimating Collision Cross Sections of Negatively Charged N-Glycans Using Traveling Wave Ion Mobility-Mass Spectrometry. *Anal. Chem.* **2014,** *86*, 10789-10795. [↑](#footnote-ref-1)
